# Mid-term outcomes of compaction autologous bone grafting in uncemented primary total hip replacement stems

**DOI:** 10.1007/s00402-024-05640-8

**Published:** 2024-12-12

**Authors:** Satomi Abe, Masahiro Inoue, Takashi Mikami, Hidefumi Honke, Masahiro Suzuki, Taiki Kanno, Takeshi Masuda

**Affiliations:** 1 Department of Orthopedic Surgery, Eniwa Hospital, 2-1-1, Koganechuou, Eniwa, Koganechuou, Hokkaido 061-1449 Japan; 2 Department of Orthopedic Surgery, Fukuoka University Faculty of Medicine, 8-19-1 Nanakuma, Jounan-ku, Fukuoka, 814-0180 Japan

**Keywords:** Compaction autologous bone grafting, Uncemented stem, Total hip replacement, CORAIL^®^ stem, Stress shielding, Reactive lines

## Abstract

**Introduction:**

Fixation and long-term stability of collared, uncemented stems, such as the CORAIL^®^ collared stem, in total hip arthroplasty (THA), depend on a strong cancellous bone sleeve and subsequent osseointegration. This study aimed to investigate bone reaction and mid-term outcomes following compaction autologous bone grafting in uncemented stems in primary THA.

**Materials and methods:**

This study retrospectively reviewed patients with primary THA using CORAIL^®^ collared stem and having ≥ 5 years follow-up. Patients were divided into the bone graft and control groups based on the use of compaction autologous bone grafting. Demographic characteristics, fracture risk, operation time, complications, revisions, and radiologic measures, such as stress shielding and reactive lines were compared between the groups.

**Results:**

A total of 140 cases (85% women, mean age: 63 years, mean follow-up: 72 months) were included. Autologous bone graft was used in 32 (23%) cases. No significant differences in terms of age, sex, diagnoses, follow-up duration, or operation time were observed between the groups. Stress shielding frequency remained stable at 9.4% between 1y and 5y in the bone graft group, but increased from 13.9 to 28.7% in the control group, resulting in the latter having a higher 5-y frequency than the bone graft group (*p* = 0.0004). Reactive lines increased from 1y to 5y in both groups (bone graft: 6.3–37.5%, *p* = 0.0015; control: 4.6–26.9%, *p* < 0.001) with no significant differences between groups. There were no instances of stem subsidence/loosening or stem revision in either group.

**Conclusions:**

Autologous compaction bone grafting achieved satisfactory fixation of the uncemented CORAIL^®^ collared stem without requiring distal fixation and mitigated stress shielding. Larger, prospective studies with longer follow-ups are needed to confirm the clinical implications of these mid-term results in primary THA.

## Introduction

Total hip arthroplasty (THA) provides long-term pain reduction and enhanced function in patients with hip osteoarthritis and is one of the most successful and cost-effective interventions in orthopedic surgery. It is estimated that over 550,000 THAs will be performed annually by 2030. Conventionally, cemented implants in THA achieved stability through mechanical interlock with bone after curing of the polymethylmethacrylate (PMMA) cement. Uncemented stems have gained significant attention and popularity in primary THR due to their long-term stability, better long-term bone preservation and reduced risk of cement-related complications and have become the most common mode of fixation employed in the US and several European countries.

In Japan, the widely used “Fit & Fill” uncemented stems face challenges, including early subsidence, stress shielding causing femoral bone atrophy, and periprosthetic fractures [1–3]. To address these issues, collared uncemented stems have been developed; in these stems, fixation and long-term stability rely on osseointegration secondary to endosteal microfractures at the time of preparation, along with subsequent bone ongrowth or ingrowth [4]. One such implant is the CORAIL^®^ stem, which has shown good results over a long period of time in Europe [5–9] and was introduced in Japan in 2013. The CORAIL^®^ collared stem compacts cancellous bone to form a cancellous bone sleeve, and is called a “silent stem” with little bone reaction [6]. However, in cases with bone fragility, the use of CORAIL^®^ collared stem makes it difficult to form a strong cancellous bone sleeve [10, 11] and therefore requires distal fixation that may result in stress shielding. We hypothesize that making a cancellous bone sleeve by compaction autologous bone grafting [12] would fix the stem, avoid distal fixation, and mitigate stress shielding. This study aimed to investigate bone reaction (stress shielding, reactive line) in bone graft cases and examine its usefulness by assessing mid-term outcomes of compaction autologous bone grafting in uncemented primary total hip replacement stems.

## Methods

### Study design

This single-center, retrospective study enrolled patients who underwent total hip arthroplasty (THA) using CORAIL^®^ collared stem (Depuy Synthes, Warsaw, IN, USA) at Wajokai Eniwa Hospital between 2013 and 2017 and had ≥ 5 years follow-up. Based on the use of additional bone graft, patients were divided into the bone graft and control (without bone graft) groups. The study was approved by Wajokai Eniwa Hospital Ethics Committee (approval no. 207), and written informed consent was obtained from all participants.

## Surgical procedure

**Fig. 1:**
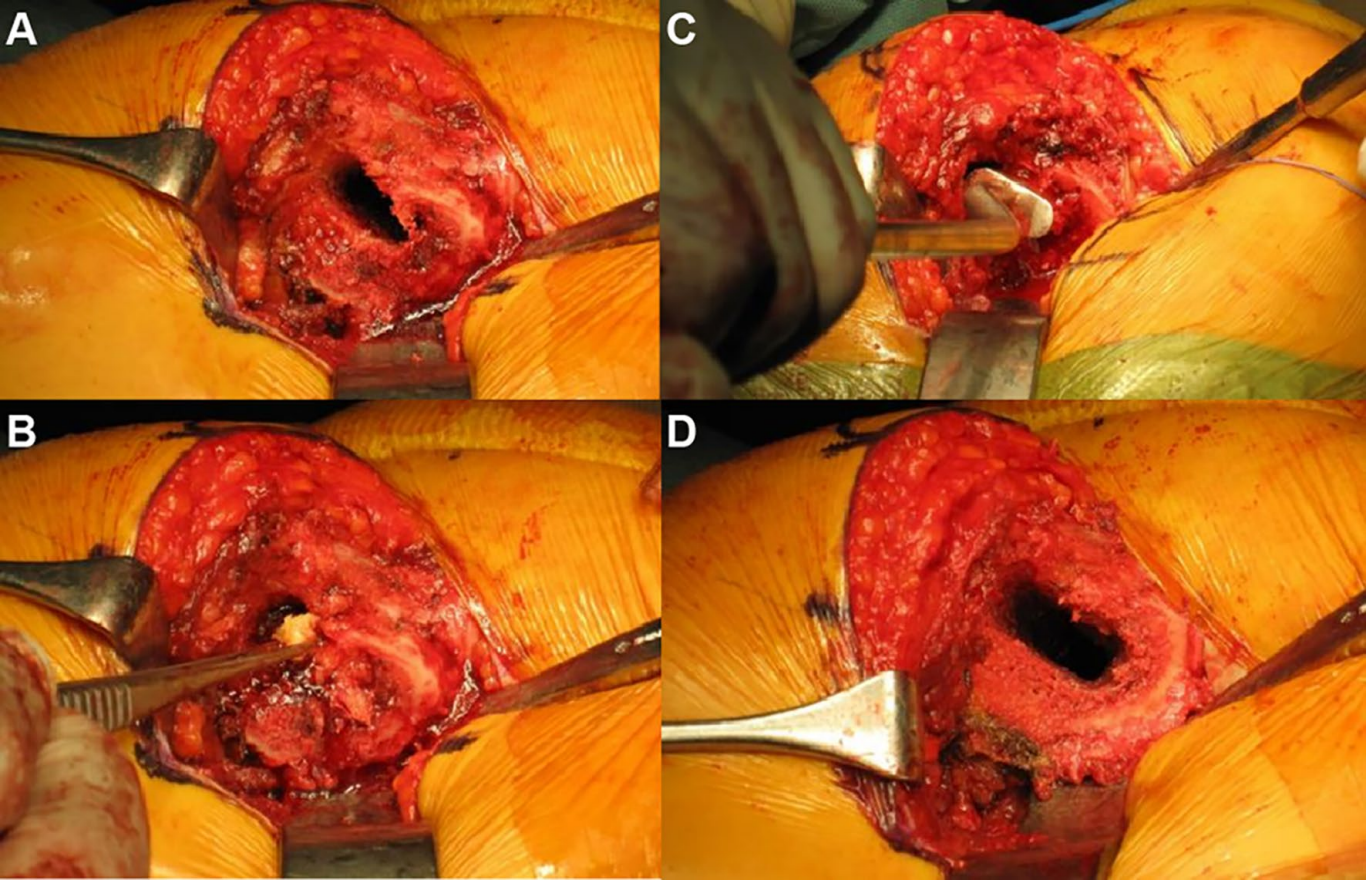
Surgical technique of using compaction bone graft prior to implantation of the uncemented CORAIL^®^ collared stem **A**, Cancellous bone sleeve before compaction bone graft **B**, Insertion of the fragmented bone grafts into the medullary canal **C**, Compaction of the bone graft with CORAIL^®^ broach or bone impactor **D**, Tough cancellous bone sleeve after compaction bone graft

All procedures were performed by two surgeons using a posterior approach. The uncemented CORAIL^®^ collared stem was fixed in the cancellous bone sleeve made by compaction broach. In cases where rotational fixation was not obtained at the planned stem size, cancellous bone fragments were collected by gnawing the femoral head using a Luer Bone Rongeur and transplanted into the femoral medullary canal to obtain fixation without increasing the stem size (Fig. 1). This technique allowed the formation of a tough cancellous bone sleeve through crushing and compression of the bone graft with the CORAIL^®^ compaction broach.

## Data extraction

Relevant data, including demographic characteristics, FRAX score for fracture risk, operation time, complications, and revisions, were extracted from electronic medical records [13, 14]. Risk factors for FRAX calculation included prior fractures, parental hip fracture history, current smoking, alcohol consumption ≥ 3 units/day, glucocorticoid use, rheumatoid arthritis, and secondary osteoporosis. FRAX scores for the risk of major osteoporosis and hip fracture were calculated.

Preoperative and postoperative hip radiographs were retrieved and assessed by the author and two other orthopedic surgeons who were not involved in the surgeries. Preoperative hip radiographs were assessed for cortical thickness index (CTI) as a measure of bone status using previously described methods [13, 14]. Postoperative X-ray images obtained at least 1 year after surgery and at final observation were assessed and compared with postoperative 1-week radiographs, and evaluated for stress shielding [15], reactive line [6], and radiolucent lines around the stem [16], using previously described methods. Femoral osteopenia resulting from stress shielding was graded as 1–4 using Engh’s classification [15]. A reactive line was defined as a parallel “linear sclerotic image” adjacent to the prosthesis. Data on complications, including stem loosening (defined as stem subsidence or the presence of radiolucent lines around the stem in postoperative X-rays), and stem revision were also extracted to represent mid-term outcomes.

### Statistical analysis

Continuous variables were tested for normality and summarized as means and standard deviation (if normally distributed), or medians and range (if not normally distributed). Categorical variables were summarized as counts and percentages. Repeated-measures ANOVA and Mann–Whitney U-tests were performed to compare the variables and outcomes between the two groups. Statistical significance was set at *p* < 0.05. Statistical analysis was performed using JMP statistical software version 17.0.0 (SAS Institute Inc., Cary, NC, USA).

## Results

### Patient characteristics

A total of 140 cases, comprising 21 men and 119 women, underwent THA using the CORAIL^®^ collared stem during the study period. Patients had an average age of 63.4 (range: 43–84) years and were followed up for 72 (60–108) months. In terms of disease diagnosis, 124 cases (89%) had hip arthritis secondary to developmental dysplasia of the hip, 8 (6%) had primary hip arthritis, 5 (4%) had idiopathic femoral head necrosis, and 3 (2%) had traumatic hip arthritis.

Autologous bone graft was used in 32 (23%) cases. The patient characteristics in the bone graft and the control groups are summarized and compared in Table 1. No significant differences in terms of age, sex, diagnoses, or follow-up duration were observed between the two groups. FRAX scores for major osteoporosis tended to be higher in the bone graft group (median, 9.7; range: 2.8–25) compared to the control group (median, 6.9; range: 1.1–32), but the difference was not statistically significant. Median operation time was 76 (43–105) minutes in the bone graft group and 71 (39–130) minutes in the control group.

**Table 1:** Characteristics of patients undergoing primary total hip arthroplasty using uncemented CORAIL^®^ collared stems with (bone graft group) or without compaction autologous bone grafting (control group)

		Total (*n* = 140)	Bone graft Group (*n* = 32)	Control Group (*n* = 108)	*p* value
Age (years)		63.4 ± 8.7	65.8 ± 9.5	62.7 ± 8.3	0.07
Sex (Male: Female) (%)		15%: 85%	12.5%: 87.5%	16.7%: 83.3%	0.57
Disease	hip arthritis secondary to developmental dysplasia of the hip	124	28	96	0.78
	primary hip arthritis	8	1	7	
	idiopathic femoral head necrosis	5	2	3	
	traumatic hip arthritis	3	1	2	
FRAX score	Major osteoporosis	7.1 (1.1–32)	9.7 (2.8–25)	6.9 (1.1–32)	0.1
	Hip Fracture	1.3 (0.1–15)	1.8 (0.2–12)	1.2 (0.1–14)	0.33
CTI		0.54 ± 0.06	0.54 ± 0.01	0.55 ± 0.01	0.54
Operation time		74 (39–130)	76 (43–105)	71 (39–130)	0.24
Follow-up duration		72 (60–108)	70 (60–97)	73 (60–108)	0.13

## Outcomes

The outcomes in the two groups are summarized and compared in Table 2. The frequency of stress shielding remained stable at 9.4% in the bone graft group at 1y and 5y, compared to an increase from 13.9% at 1y to 28.7% at 5y in the control group (*p* < 0.001). At 5y, the bone graft group had a significantly lower frequency of stress shielding compared to the control group (*p* = 0.0004), with no cases having grades 3 or 4 stress shielding in the bone graft group.

**Table 2:** Study outcomes at 1y and 5y following primary total hip arthroplasty using uncemented CORAIL^®^ collared stems with (bone graft group) or without compaction autologous bone grafting (control group). (n, %)

		Bone graft Group (*n* = 32)	Control Group (*n* = 108)	*p* value
Stress shielding				
(Grade 1, 2, 3, 4)	(1y)	3 (9.4%) (1, 2, 0, 0)	15 (15%) (9, 6, 0, 0)^#^	0.24
	(5y)	3 (9.4%) (1, 2, 0, 0)	31 (28.7%) (13, 16, 2, 0)	0.0004*
Reactive lines	(1y)	2 (6.3%)	5 (5%)^#^	0.33
	(5y)	12 (37.5%)	29 (26.9%)	0.31
Stem loosening		0	0	NA
Stem revision		0	0	NA

^#^1-year X-rays were missing in 7 cases

**Fig. 2:**
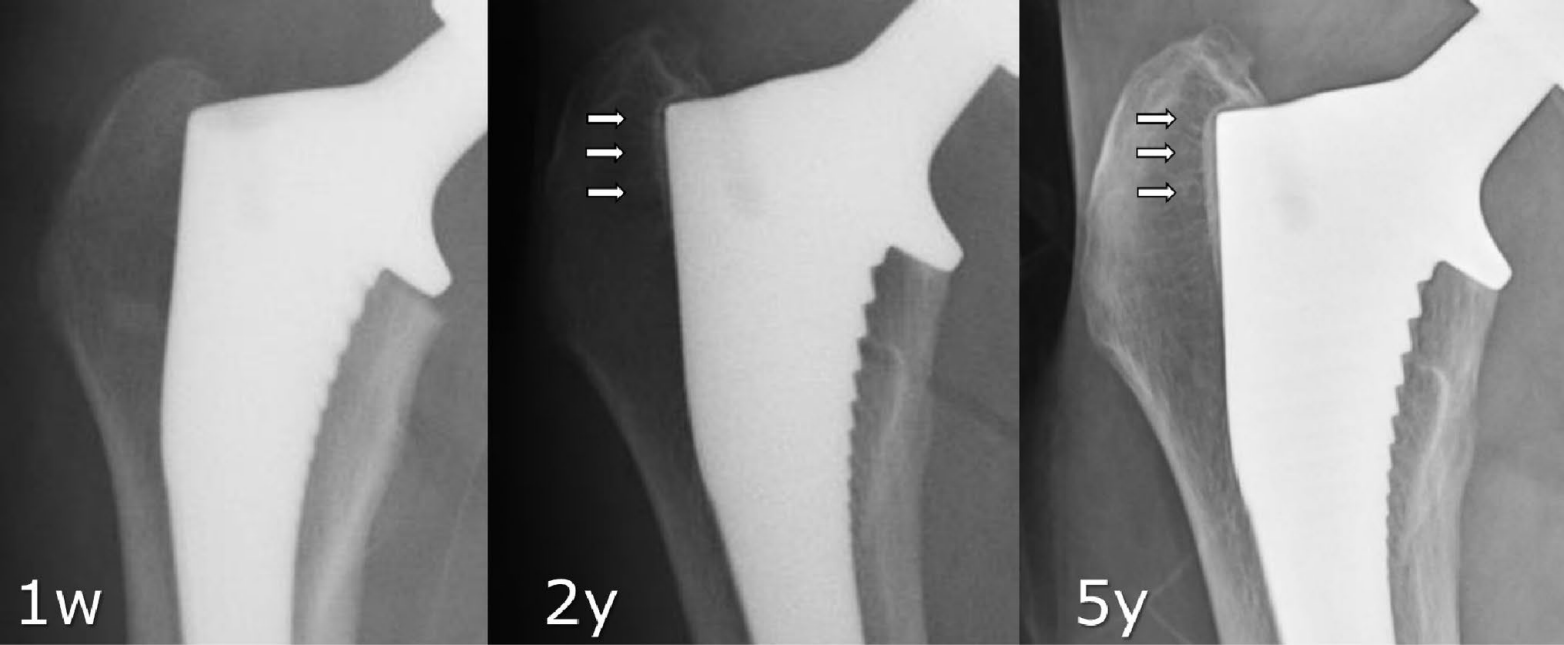
Evidence of reactive lines (arrows) in longitudinal hip radiographs in a patient who had THA using CORAIL^®^ collared stem without bone graft (control group)

**Fig. 3:**
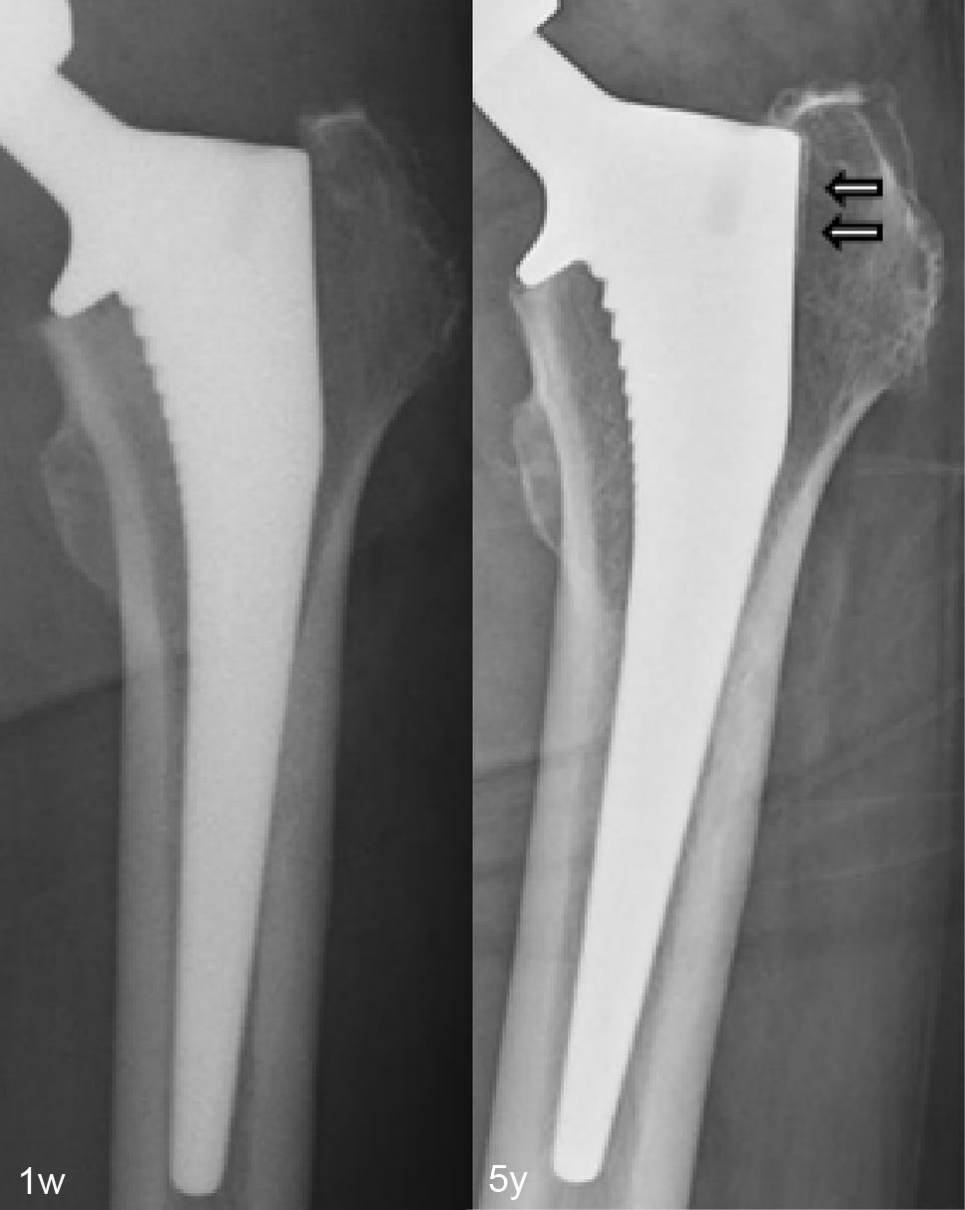
Longitudinal hip radiographs of an 81-year-old female patient who had THA using CORAIL^®^ collared stem with bone graft showing evidence of reactive lines (arrows), but no stress shielding, 5 years after surgery

**Fig. 4:**
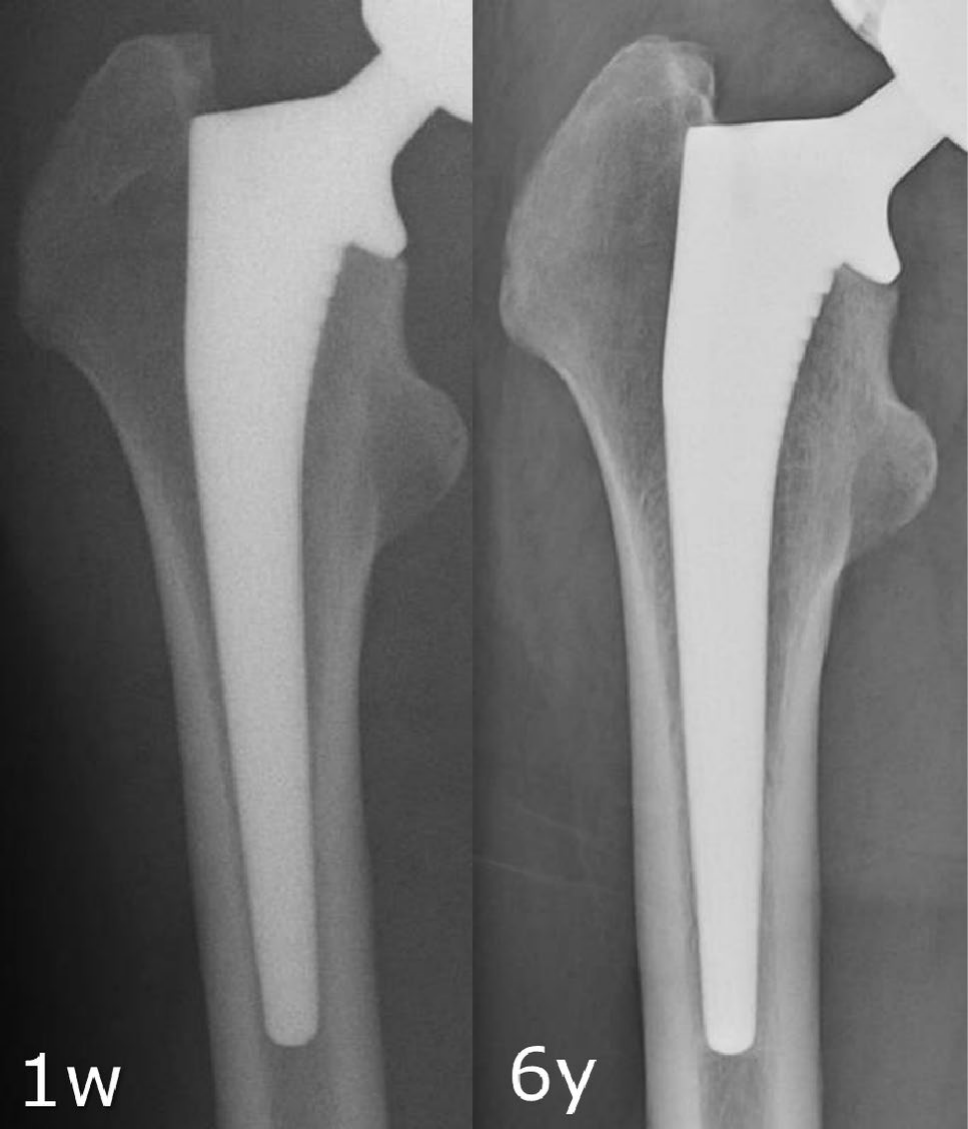
Longitudinal hip radiographs of a 55-year-old male patient who had THA using CORAIL^®^ collared stem with bone graft showing no evidence of reactive lines or stress shielding, 6 years after surgery

Reactive lines increased in frequency from 1y to 5y in both groups (6.3–37.5% in the bone graft group, *p* = 0.0015; 4.6–26.9% in the control group, *p* < 0.001, Fig. 2), but no statistically significant differences were observed between the two groups at either time point. As an example, Fig. 3 shows X-rays of an 81-year-old woman with THA using CORAIL^®^ collared stem and compaction bone graft demonstrating evidence of reactive lines, but no stress shielding, 5 years after surgery. Figure 4 shows X-rays of a 55-year-old man with a bone graft who had no evidence of reactive lines or stress shielding 6 years after surgery. There were no instances of stem subsidence/loosening or stem revision in either group during the study period.

## Discussion

Stress shielding is argued to be the most important mechanism causing periprosthetic defects in the femur [12]. Furthermore, aseptic loosening of femoral stems is one of the most common causes of implant failure, and stress shielding is the primary mechanical factor causing aseptic loosening [17]. Therefore, it is important to reduce stress shielding to achieve good long-term results. This study aimed to assess mid-term outcomes following the use of compaction autologous bone grafting with uncemented CORAIL^®^ collared stems in primary total hip arthroplasty, particularly investigating bone reaction (stress shielding, reactive line) in cases with and without bone graft. As hypothesized, the compaction autologous bone grafting technique achieved effective stem fixation without necessitating distal fixation and mitigated stress shielding.

Patient characteristics, including age, sex, diagnosis, FRAX score, or CTI, did not significantly differ between the groups, although FRAX scores tended to be higher in the bone graft group (median, 9.7) compared to the control group (median, 6.9). A cemented stem is usually selected when preoperative X-rays show evidence of bone weakness. Notably, there were several cases where bone fragility was not expected from plain X-rays, but was encountered intraoperatively when the cancellous bone was broached; autologous bone graft was used in such cases. This may explain the small difference in CTI and FRAX scores between the two groups. Further, this indicates that bone grafting was not preferentially used in cases with more severe pathology (e.g., advanced age or osteoporosis). Additionally, the compaction bone graft procedure did not significantly extend operation time, supporting its feasibility in establishing a cancellous bone sleeve before uncemented CORAIL^®^ collared stem implantation during primary total hip arthroplasty.

In a previous study [12] that used a CORAIL^®^ collarless stem combined with bone grafting, one patient in the graft group and three patients in the no-graft group had more than 3 mm of subsidence at 2 years postoperative follow-up. In addition, one patient in the graft group had a revision of the femoral component at 4 months postoperatively due to loosening of the prosthesis caused by the small size of the stem, and one patient in the no-graft group had one intra-operative calcar clack. On the other hand, this study showed excellent mid-term clinical outcomes with no cases of stem loosening and revision in either group during an average follow-up of 75.1 months. These suggest that the CORAIL^®^ collared stem generally achieves good fixation with or without compaction bone grafting.

Longitudinal radiographic analysis showed a gradual increase in the frequency of both stress shielding (13.9–28.7%) and reactive lines (4.6–26.9%) in the control group over five years. On the other hand, while reactive lines increased in frequency (6.3–26.9%) in the bone graft group over five years, the frequency of stress shielding remained stable (9.4%) and was significantly lower than in the control group at 5 years. These results suggest that compaction bone grafting offers some mechanical advantage by preventing stress shielding while achieving stable fixation in CORAIL^®^ collared stems, though the clinical benefits are not apparent over the mid-term. Longer follow-up may be required to understand if bone grafting has any clinical benefit in the long term.

The use of bone graft was not associated with any other bone changes, as suggested by the absence of significant differences in reactive lines in the two groups. This could be explained by the fact that there was no difference between the two groups because the greater trochanter is the only site subjected to tensile and sliding stresses, not compressive stress, and is not affected by the collar or bone grafting.

Our results are in agreement with that from a previous study [18] that used bone grafting into the femoral medullary canal for uncemented AML^®^ stem in 110 cases and achieved good stem fixation in 99% of cases, suggesting the possibility of reducing stress shielding by using a more appropriate size. In addition, the use of appropriately sized stems may also reduce revision surgeries due to stem loosening or fracture.

The study has some limitations. First, this was a single-center retrospective study with a small sample size. Larger multi-center studies may be needed to confirm these results. Second, though the two groups did not show any difference in demographic and disease variables that we assessed, there may be differences in variables that were not evaluated in the study, and these might have confounded the results. A prospective, randomized study or the use of multivariable analysis that adjusts for potential confounders may be needed to avoid possible bias in the current study design and obtain more definitive results. Finally, only mid-term outcomes were investigated in this study and clinical outcomes generally become apparent over the long term in patients with THA. Studies with longer follow-ups are needed to confirm the clinical implications of the mid-term clinical and radiologic observations from the current study.

In summary, our study demonstrates that autologous compaction bone grafting not only achieves satisfactory fixation of the uncemented CORAIL^®^ collared stem at an appropriate size but also reduces the incidence of stress shielding, indicating its potential benefit in primary total hip replacement. Larger, prospective studies with extended follow-ups are essential to validate the clinical implications derived from the mid-term clinical and radiologic results presented in this study.

## Data Availability

Data sharing is not applicable to this article as no datasets were generated or analyzed during the study.
